# Clinical evaluation of the first domestically produced generic isosulfan blue injection in sentinel lymph node biopsy for early breast cancer in China: a multicenter, single-arm, open validation trial (CSBrS-024)

**DOI:** 10.1097/CM9.0000000000002309

**Published:** 2022-08-11

**Authors:** Fengjiang Qu, Di Wu, Yunjiang Liu, Wei Gao, Shu Wang, Siyuan Wang, Feng Jin, Bo Chen, Jian Huang, Shanshan Sun, Zhimin Fan

**Affiliations:** 1 Breast Surgery Department, General Surgery Center, The First Hospital of Jilin University, Changchun, Jlilin 130021, China; 2 The Breast Center, the Fourth Hospital of Hebei Medical University, Shijiazhuang, Hebei 050011, China; 3 Department of Breast Surgery, Peking University People's Hospital, Beijing 100044, China; 4 Department of Breast Surgery, the First Hospital of China Medical University, Shenyang, Liaoning 110001, China; 5 Department of Breast Surgery, Key Laboratory of Tumor Microenvironment and Immune Therapy of Zhejiang Province, Second Affiliated Hospital, Zhejiang University School of Medicine, Hangzhou, Zhejiang 310009, China.

In 2020, the first generic isosulfan blue product for injection (notification number: 2020 LP00901, batch number: 118210301, 5 mL: 50 mg, Guangdong Hebo Pharmaceutical Co., Ltd.) was approved by the National Medical Products Administration of China. This multicenter, single-arm, phase III, open clinical validation trial was organized by the Chinese Society of Breast Surgery to evaluate the efficacy and safety of this domestically produced generic isosulfan blue injection for sentinel lymph node biopsy (SLNB) in patients with early breast cancer (BC) in China.

***Inclusion criteria.*** (1) A full understanding of the purpose, nature, and methods of the study, voluntary participation, and signing of the informed consent form; (2) age 18 to 70 years, of either sex; (3) histopathological diagnosis of invasive BC or carcinoma *in situ*, with the clinical Tumor-Node-Metastasis stage of cT_1–2_N_0_M_0_; (4) no history of radiotherapy on the breast or chest wall; (5) no surgical contraindications on preoperative examination; (6) ability to maintain good communication with the investigator and understand and comply with the requirements of the clinical trial.

***Exclusion criteria.*** (1) Allergy to the investigational drug or its excipients, triphenylmethane or related compounds, or other dyes, or a history of allergies to two or more drugs, foods, etc; (2) severe or uncontrollable disease, such as severe cardiovascular or cerebrovascular disease (hematological disease, chronic congestive heart failure with the New York Heart Association grade ≥ III, etc), severe pulmonary insufficiency, psychosis, bronchial asthma, and allergy; (3) surgery or neoadjuvant chemotherapy for BC on the affected side; (4) any form of breast augmentation surgery; (5) liver or kidney dysfunction; (6) current pregnancy or breastfeeding or a pregnancy plan that made the patient unwilling to take effective contraceptive measures, or a plan to donate eggs from the screening period to within 3 months after drug administration; (7) positivity for any of the indicators in the infectious disease screen; (8) participation in any other clinical trial within the past 3 months.

***Steps of the SLNB procedure*** [Supplementary Figure 1]. Within 5 min before the operation, the first domestically produced generic isosulfan blue injection (total dose ≤3 mL) was subcutaneously injected into the mammary areola or the periphery of the mass at 1 to 3 points. After massage, the skin was incised. The surgeon visually located and photographed the blue-stained lymphatic vessels and blue-stainedsentinel lymph nodes (SLNs). The total number of resected SLNs and the number of blue-stained SLNs were recorded in the pathology department, and the surgical procedure for axillary lymph node dissection (ALND)^[1]^ was selected based on the pathological report. The vital signs and hematologic indicators of the subjects were monitored 1 h (±0.5 h) before drug administration, during the operation, 3 days after the operation, and during the safety follow-up period (14 ± 2 days after drug administration). The occurrence and treatment of adverse events were monitored and recorded throughout the trial period.

***Statistical methods.*** Statistical analysis of the population: The efficacy and safety (endpoint indicators) were evaluated based on the per-protocol set. Statistical analysis was performed using SAS (version 9.4, Inc., Cary, NC, USA). Descriptive statistical methods were used for quantitative and count indicators. The one-sided significance level was at 0.025.

A total of 150 patients with cT_1–2_N_0_M_0_ were enrolled in this study, all of whom were female, aged 21 to 70 years, with a median age of 50 years. Pathological types: There were 123 cases (82.0%) of invasive BC, 27 cases of carcinoma *in situ* (18%). Surgical procedures: There were 53 cases (35.3%) of breast-conserving surgery, 91 cases (60.7%) of simple mastectomy plus SLNB, and six cases (4.0%) of nipple–areola complex–sparing mastectomy and immediately reconstruction. A total of 117 patients (78.0%) received SLNB, and 33 SLNB-positive patients (22.0%) received completion ALND.

Success rate of lymphatic vessel detection and the accuracy of SLN detection in SLNB after generic isosulfan blue injection in these 150 patients with early BC [Table 1].

**Table 1:** Success rate of lymphatic vessel detection and success rate and accuracy of SLN detection in 150 patients with early BC who received SLNB using the generic isosulfan blue injection.

Experimental measure	Per-protocol set
Success rate of lymphatic vessel detection^∗^	
Number of successful cases of lymphatic vessel detection	147
Success rate (%)	98.0
Success rate of SLN detection^†^	
Patients with ≥1 blue-stained SLN detected	141
Success rate (%)	94.0
Accuracy of SLN detection^‡^	
Number of blue-stained SLNs detected in SLNB	337
Number of blue-stained SLNs confirmed finally in pathological sections	382
Number of nonblue-stained SLNs confirmed finally in pathological sections	16
Accuracy (%)	96.0
Sensitivity (%)	100

∗ Success rate of lymphatic vessel detection = Number of successful cases of lymphatic vessel detection/number of cases administered with the tracer × 100%. Lymphatic vessel detection was determined to be adequate or inadequate by the clinician based on intraoperative visual observation, and an adequate blue-stained lymphatic vessel was considered successful detection.

† Successful SLN detection was defined as the detection of ≥1 blue-stained SLN. Success rate of SLN detection = number of successful SLN detection cases/number of cases given the tracer × 100%.

‡ Accuracy of SLN detection = Number of blue-stained SLNs confirmed by pathological sections stained with the tracer/(number of blue-stained SLNs confirmed by pathological sections stained with the tracer + number of nonblue-stained SLNs confirmed by pathological sections stained with the tracer) × 100%. Only the number of blue-stained SLNs detected in SLNB and the numbers of SLNs and nonblue-stained SLNs that were confirmed by pathological sections were collected. Based on the statistical criteria of the screening test and the obtained data, the number of blue-stained SLNs was confirmed accurate in pathological sections. The sensitivity and false-negative rate can be calculated, but with a certain deviation. No nonblue-stained non-SLN was found in any pathological section, so the true-negative rate could not be obtained. Consequently, the specificity and false-positive rate could not be calculated. BC: Breast cancer; CI: Confidence interval; SLN(s): Sentinel lymph node(s); SLNB: Sentinel lymph node biopsy.

A total of 41 adverse events occurred in 35 patients (23.3%), including three serious adverse events in three patients (2.0%) [Supplementary Table 1].

***Discussion.*** SLNB is a well recognized, standard surgical procedure to assess the axillary status of patients with clinically axillary-negative BC.^[1]^ Commonly used SLNB tracers include blue dyes (patent blue, isosulfan blue, methylene blue, etc), radionuclides, nanocarbon, and fluorescent dyes (indocyanine green). Methylene blue is the most commonly used tracer in China due to its low cost and high accessibility. The detection rate and false-negative rate of SLNB have been clinically confirmed.^[2]^ Patent blue is an isomer of isosulfan blue. The detection rate of SLNB using patent blue alone has been 83.2%,^[3]^ but patent blue has not been marketed in China. The clinical application of radionuclides is limited because radionuclide management (disposition, processing, and preparation) must be performed by qualified physicians. Nanocarbon is also a lymphatic tracer for clinical application. The detection rate and false-negative rate of SLNB using nanocarbon was 99.1% and 4.1%, respectively,^[4]^ though the clinical application of nanocarbon is hampered by its high price and permanent pigment deposition due to the difficulty of metabolizing nanocarbon. The detection rate of SLNB using indocyanine green alone was 97%, and the detection rate of SLNB using indocyanine green plus blue dye reached 99.6%.^[5]^ However, indocyanine green imaging requires special fluorescence imaging equipment, which is cumbersome to operate and difficult to apply widely in hospitals.

In contrast, isosulfan blue injection has the advantages of simple and convenient use, high detection rate, and high accuracy so it has been widely used as an SLNB tracer in many countries. A prospective randomized controlled trial compared the differences between the use of a radionuclide plus isosulfan blue *vs.* radionuclide alone to trace SLNs in cN_0_BC patients, and the results showed that there was no difference in the detection rate between the two (*P* = 0.58).^[6]^ The endpoints of a phase III clinical trial were the effectiveness and safety of lymphatic detection, with adequate and inadequate detections of lymphatic vessels as indicators to evaluate the effectiveness, and the results showed that isosulfan blue is a reliable tracer for lymphatic vessel detection in SLNB.^[7]^

The results of this study showed that the success rate of lymphatic vessel detection with the first domestically produced injectable generic isosulfan blue in China was 98.0%, and the success rate and the accuracy of SLN detection were 94.0% and 96.0%, respectively, which were superior to the results of existing studies. In this study, we found that using the first domestically produced generic isosulfan blue injection alone as the tracer for SLNB, two or more SLNs were detected in 94% (141/150) of the patients, a total of 382 blue-stained SLNs were finally confirmed in pathological sections. The results suggest that more SLNs could be detected using the first domestically produced generic isosulfan blue injection in China, with high accuracy.

Like other blue dye tracers, isosulfan blue has the risk of allergic reactions, including spontaneously resolved erythema and urticaria, though some patients need intensive care unit treatment.^[8]^ There is nothing to do with isosulfan blue because these complications such as fever, difficulty urinating, wound inflammation, and so on are also observed in routine BC surgery and anesthesia. Three serious adverse events occurred in three patients (2.0%), including one case (0.7%) of deep venous thromboembolism (VTE), one case (0.7%) of cerebral infarction, and one case (0.7%) of postoperative wound infection. The incidence of VTE after breast surgery is relatively low.^[9]^ In this study, the incidence of VTE was 0.7%, and the drug administration was already done when VTE occurred, so the VTEs might not have been related to the investigational drug. One patient had underlying diseases that were perioperative risk factors, namely, hypertension and coronary heart disease, before the operation. On the 4th day after discharge, the patient had mobility and language impairment and was diagnosed with cerebral infarction, which improved after symptomatic treatment. This case of cerebral infarction was related to the underlying diseases and long-term atherosclerosis. The investigational drug is physiologically inert, has no pharmacologic effect, and cannot enter the blood through the blood vessel wall. Hence, this adverse event was definitely not related to the investigational drug. Another patient developed an incision infection on the 5th day after the operation. After antibiotics and regular incision cleaning and dressing changes, the patient recovered. This adverse reaction was also not related to the investigational drug because the incision became infected long after drug administration (the drug is excreted through the urine within 24 h of drug administration). In summary, the three serious adverse events observed during this study were definitely not related to the use of the investigational drug.

The results of this multicenter, single-arm, open validation test show that the first domestically produced generic isosulfan blue injection in China as an SLNB tracer has a good success rate and accuracy of lymphatic vessel detection and SLN detection, with few side effects and spontaneously resolved mild symptoms. The generic isosulfan blue injection has the advantages of ease of use, high convenience, and good accessibility, and it is safe and effective for SLNB in Chinese patients with early BC.

## Supplementary Material

Supplemental Digital Content
