# Supplementary material for: Clinical evaluation of the first domestically produced generic isosulfan blue injection in sentinel lymph node biopsy for early breast cancer in China: a multicenter, single-arm, open validation trial (CSBrS-024)
Source: Chin Med J (Engl). 2022 Aug 11;135(14):1753–5. doi: 10.1097/CM9.0000000000002309 (PMC9509065; doi:10.1097/CM9.0000000000002309)
Supplement: Supplemental Digital Content [file cm9-135-1753-s001.pdf]

Sentinel lymph node biopsy (SLNB) is a well-recognized standard surgical procedure to assess the axillary status of patients with clinically axillary-negative early breast cancer (BC). Patients with negative sentinel lymph nodes (SLNs) have the same prognosis as patients with negative axillary lymph node dissection (ALND).[1–4] Blue dyes are the most commonly used lymphatic tracers in clinical practice. Among them, patent blue, isosulfan blue, and methylene blue have widely recognized clinical value. In 1981, the United States Food and Drug Administration approved the first staining dye, isosulfan blue ($C_{27}H_{31}N_2NaO_6S_2$) for injection for lymphangiography. The subcutaneously injected isosulfan blue injection is selectively and rapidly absorbed by the lymphatic vessels through local penetration. Isosulfan blue injection has been widely used in SLNB in countries outside China because of its non-pharmacological features, such as ease to use, rapid effectiveness, and visibleness. The original product LYMPHAZURIN has been off the market because the raw materials were out of production, and the internationally used isosulfan blue injection is the generic product MYLAN in most countries. However, clinical application of isosulfan blue injection has not been reported in China.

Participating institutions:

The participating institutions were The First Hospital of Jilin University, The Fourth Hospital of Hebei Medical University, Peking University People's Hospital, The First Hospital of China Medical University, and The Second Affiliated Hospital of Zhejiang University School of Medicine. The trial has been approved by the ethics committee of each participating institution.

The limitation of this study is that only numbers of blue-stained SLNs, pathology-confirmed blue-stained SLNs and non-stained SLNs are collected. In fact, patients with negative SLN or 1–2 positive SLN, who meet the Z0011 trial standard did not undergo completion ALND. Thus, we

could not acquire the comprehensive status of axillary lymph nodes, which will lead to some deviations in terms of sensitivity and false-negative rates, as well as unobtainable specificity and false-positive rates.

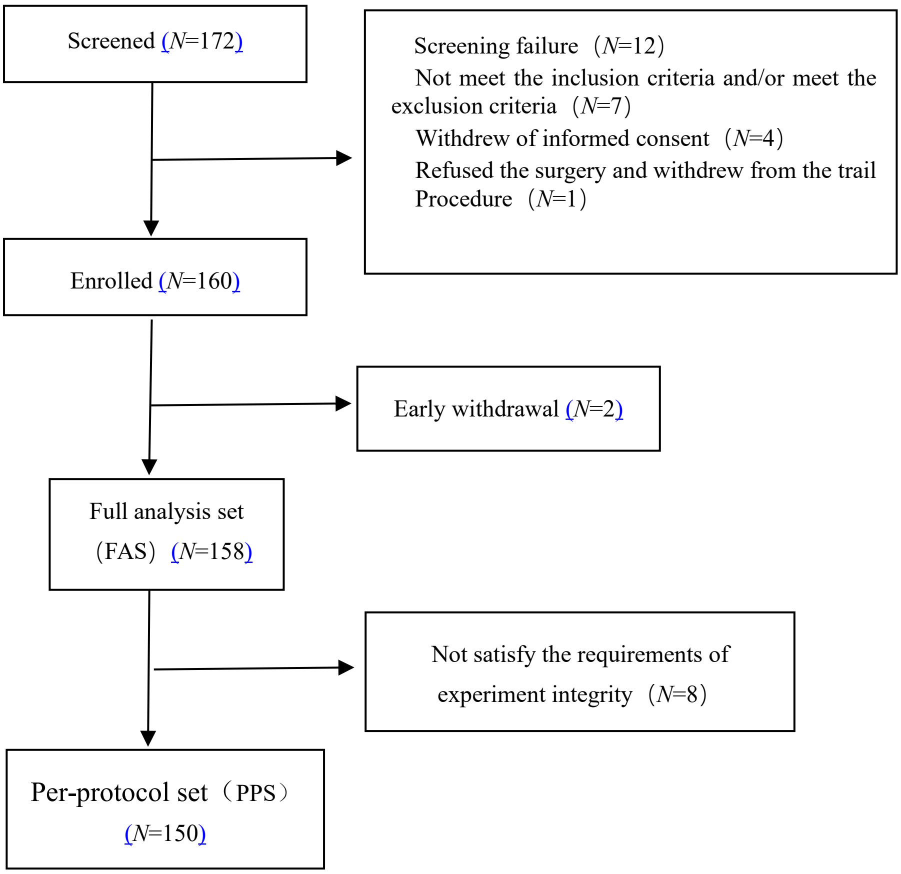

Figure 1: Flowchart of subject inclusion into the trial of the first domestically produced generic isosulfan blue injection in China.

Table S1: Occurrence of adverse reactions in 150 patients with early BC who received SLNB using generic isosulfan blue injection.

	**Per-protocol set (N = 150 patients)**	
**Adverse events**	**Number of cases**	**Number of patients (%)**
Fever	10	10 (6.7)
Difficulty urinating	9	9 (6.0)
Deep VTE	4	3 (2.0)
Thrombosis	1	1 (0.7)
Wound inflammation	4	4 (2.7)
Postoperative wound infection	2	1 (0.7)
Urinary tract infection	2	2 (1.3)
Elevated blood bilirubin	1	1 (0.7)
Elevated white blood cell count	1	1 (0.7)
Hypokalemia	3	3 (2.0)
Anemia	1	1 (0.7)
Palpitations	1	1 (0.7)
Nausea	1	1 (0.7)
Cerebral infarction	1	1 (0.7)
Total	41	35 (23.3)

BC: Breast cancer; SLNB: Sentinel lymph node biopsy; VTE: Venous thromboembolism.
